# Valvular Heart Disease Presenting as Sympathetic Crashing Acute Pulmonary Edema (SCAPE) Phenomenon: A Diagnostic and Management Paradigm

**DOI:** 10.7759/cureus.32352

**Published:** 2022-12-09

**Authors:** Harish Ashok, Jurgen Shtembari, Eliz Achhami, Suman Gaire, Dhan B Shrestha, Tilak Joshi

**Affiliations:** 1 Department of Internal Medicine, Ross University School of Medicine, Bridgetown, BRB; 2 Department of Internal Medicine, Mount Sinai Hospital, Chicago, USA; 3 Department of Internal Medicine, Sukraraj Tropical & Infectious Disease Hospital, Kathmandu, NPL

**Keywords:** nitroglycerin, bipap, valvular heart disease, acute heart failure, sympathetic crashing acute pulmonary edema

## Abstract

Sympathetic crashing acute pulmonary edema (SCAPE) is an acute decompensated heart failure due to sympathetic overflow. SCAPE is usually triggered by acute insults with an underlying substrate such as long-standing hypertension, chronic heart failure, and valvular heart disease. We present a case of SCAPE in a 91-year-old female due to underlying multivalvular heart disease. Because of severe acute presentation, SCAPE should be identified early, and management should be urgently done to decrease the need for invasive ventilation and prolonged hospitalization.

## Introduction

Sympathetic crashing acute pulmonary edema (SCAPE) is an acute presentation of heart failure (HF) resulting due to sympathetic overactivity [1]. The catecholamine surge causes a significant increase in arterial constriction and the development of flash pulmonary edema regardless of volume status. The underlying pathophysiology of SCAPE is a change in afterload due to sympathetically driven vasoconstriction and hypertension [2]. SCAPE usually results from the combination of an underlying disease such as HF, valvular heart disease, and renal failure in the presence of an acute trigger. Chronic left ventricular failure predisposes patients to SCAPE [2]. This case report describes a case of valvular heart disease with SCAPE, which was treated with positive pressure ventilation and intravenous nitroglycerin infusions.

## Case presentation

A 91-year-old female patient presented to the emergency department with bilateral lower limb edema and sudden onset of shortness of breath (SOB) while lying in bed. She also reported a non-productive cough for the last three days but denied hemoptysis, chest pain, abdominal pain, nausea, vomiting, diarrhea, or lower extremity pain. Emergency medical services (EMS) found her in a hypoxic state with an oxygen saturation of 50% on room air. While en route to the hospital, the patient received oxygen using a non-rebreather (NRB) mask, which improved pulse oximetry to 90%.

She had a 10-year history of hypertension, which was treated with hydrochlorothiazide and nifedipine. She also gave a history of bilateral breast carcinoma, diagnosed in December 2020, requiring radiation and chemotherapy, and subsequently underwent a bilateral mastectomy. In addition, the patient had a history of deep venous thrombosis and had undergone partial left thyroidectomy.

On examination in the emergency room, she was afebrile, hypertensive with a blood pressure of 156/88 mmHg, tachypneic with a respiration rate of 39 breaths/minute, tachycardic with a heart rate of 130 beats/minute, and was on NRB mask maintaining optimum saturation. On chest auscultation, bilateral breath sounds with crackles and wheezes were present in the lung bases. In addition, the patient had bilateral lower extremity pitting edema (2+). The patient was transitioned from an NRB mask to bi‑level positive airway pressure (BiPAP) and received sublingual nitroglycerin. The patient subsequently received intravenous (IV) nitroglycerin.

**Figure 1:**
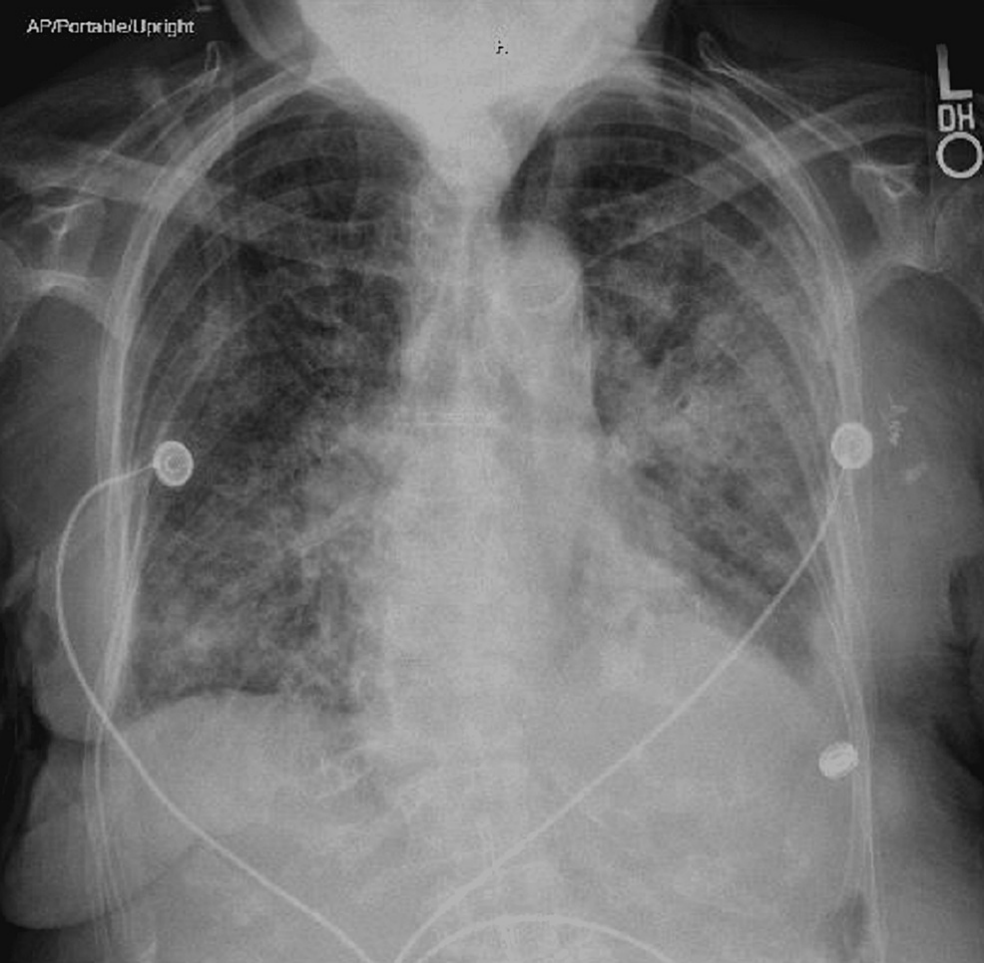
Chest X-ray showing diffuse multifocal interstitial opacification with cardiomegaly and pulmonary edema

**Figure 2:**
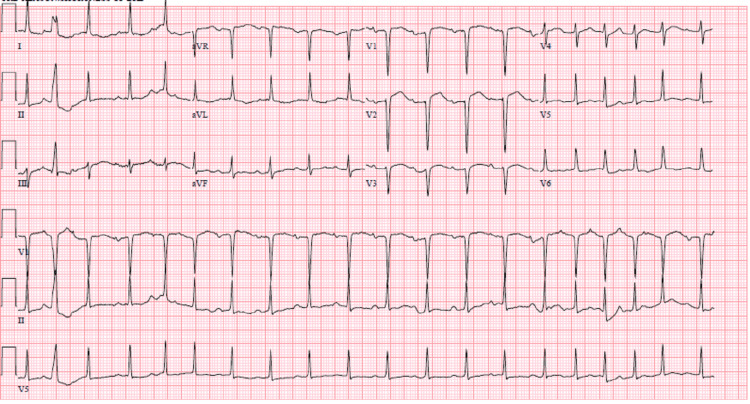
EKG taken at presentation in the emergency department showing atrial fibrillation with rapid ventricular rate EKG: electrocardiogram.

Chest X-ray demonstrated diffuse multifocal interstitial opacification throughout the lungs with cardiomegaly and pulmonary edema in the background (Figure 1). Her significant initial laboratory results are shown in Table 1. Potassium and magnesium were supplemented through an IV route for the goal of above 4 mmol/L and 2 mmol/L, respectively. A repeat electrocardiogram (EKG) showed atrial fibrillation with a rate of 118 beats per minute with no ST-elevation and T wave inversions (Figure 2). The patient reported improvement in symptoms throughout her treatment in the ED. In ED, the patient was alert and oriented to person, place, and time, resting comfortably on a BiPAP setting at 60% oxygen. IV nitroglycerin was stopped, and IV furosemide 40 mg was started.

**Table 1:** Significant initial laboratory panel mmol/L: millimoles per liter; pg/mL: picograms per milliliter; ng/ml: nanograms per milliliter.

Laboratory parameters	Lab value	Reference range
Magnesium	1.8 mmol/L	1.6-2.6
Potassium	3.0 mmol/L	3.2-5-2
Brain natriuretic peptide	1732 pg/mL	1-100
High-sensitive troponin I	67 ng/ml	0-12
Hemoglobin A1C (HbA1c)	6.1%	<5.6

**Figure 3:**
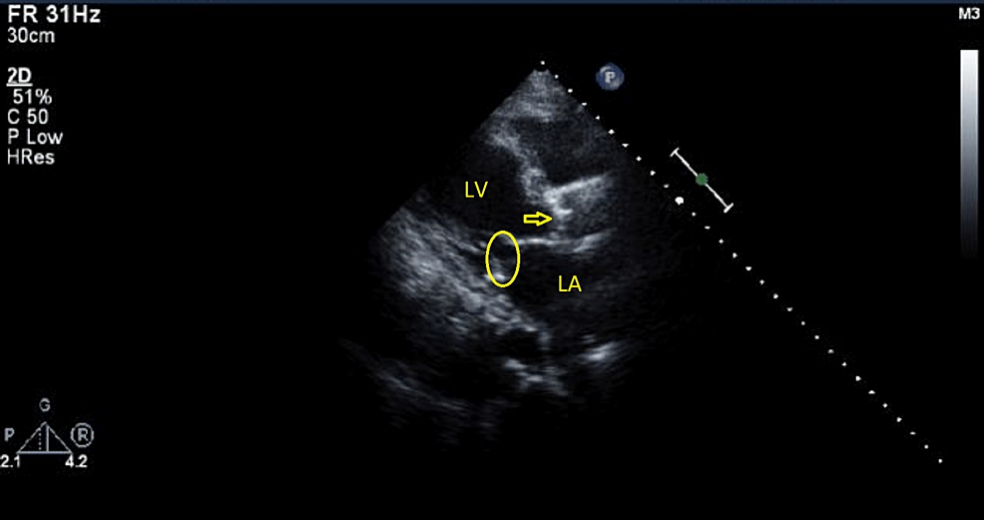
2D ECHO image showing the opening of MV and closed AV with thickening of MV leaflets 2D ECHO: two-dimensional echocardiography; MV: mitral valve; AV: aortic valve; LA: left atrium; LV: left ventricle.

**Figure 4:**
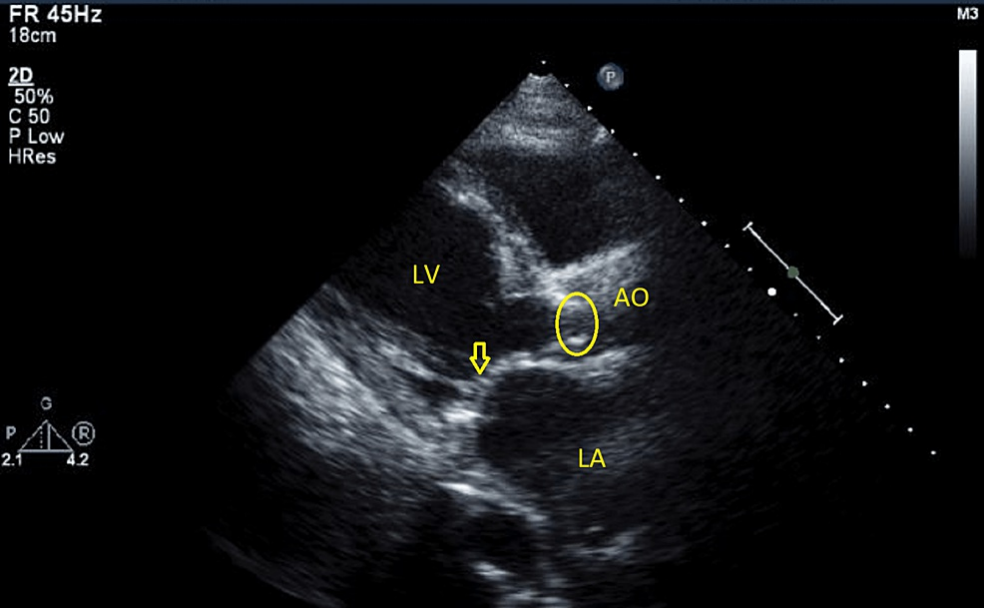
2D ECHO showing closed MV and open AV 2D ECHO: two-dimensional echocardiography; MV: mitral valve; AV: aortic valve; LA: left atrium; LV: left ventricle.

**Figure 5:**
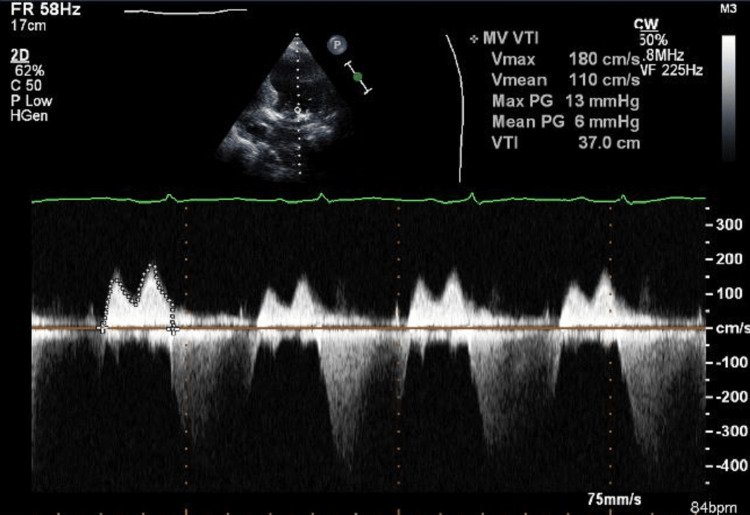
2D ECHO Doppler image showing features suggestive of MS 2D ECHO: two-dimensional echocardiography; MS: mitral stenosis.

On the next day, she reported improvement in her symptoms. A holosystolic murmur and crepitations with audible expiratory wheezes were noted on chest auscultation. The vascular duplex ultrasound of the bilateral lower limbs showed eccentric echogenic densities relating to fibrin stranding without clear thrombus, from chronic deep venous thrombosis. There was no definitive evidence of acute occlusive deep venous thrombosis. The echocardiography demonstrated normal left ventricle cavity size with an ejection fraction of 30-35%. The left atrium was markedly dilated, while the right atrium was mildly dilated. The mitral valve annulus was moderately calcified with moderately thickened leaflets (Figure 3). There was severe mitral stenosis (MS), with an area of 1.2 cm2 and a mean pressure gradient of 6 mmHg (Figures 4, 5). There was also mild mitral regurgitation (MR), directed posteriorly. The aortic valve demonstrated low-flow, low-gradient severe aortic stenosis (AS) versus pseudo severe stenosis, with a calculated area of 0.8 cm2 (0.5 cm2/m2), mean pressure gradient of 13 mmHg, and Vmax was 2.5 m/s.

The patient improved symptomatically with the management and was later discharged to cardiac rehabilitation, as the patient and family opted for conservative management for MS and AS given her age and comorbidities.

## Discussion

Acute heart failure syndrome (AHFS) contributes to a significant proportion of patients visiting the emergency room with complaints of dyspnea globally. It is more common in older patients, patients with a history of hypertension, and those with a preserved ejection fraction. The presentation of AHFS may range from mild pulmonary edema to severe cardiogenic shock. Patients with SCAPE have severe AHFS. It presents with an abrupt onset and symptoms, which quickly progress to flash pulmonary edema; therefore, these patients have a short window of time to treat appropriately to reduce their mortality and hospital stay [1].

The pathophysiology for the development of heart failure symptoms in SCAPE includes a sympathetic surge-driven increase in afterload (systemic vasoconstriction) and a lowering of venous capacitance [2,3]. SCAPE results in pulmonary edema because of fluid redistribution from peripheral to pulmonary circulation. Chronic hypertension results in a reduction of vascular compliance and smooth muscle hypertrophy. Because of the reduction in vascular compliance, the resistance to left ventricular (LV) forward flow is increased. Increased afterload in the setting of impaired ventricular compliance leads to an imbalance and ventricular-vascular coupling [3]. SCAPE usually results from the combination of an underlying disease in the presence of an acute trigger [2]. Chronic LV failure and a history of hypertension are common underlying conditions [4]. The typical acute triggers may include non-adherence with antihypertensive, volume overload, sympathomimetic intoxication, acute myocardial infarction, exertion, stress or anxiety, and acute valve dehiscence [2]. In the present case, the patient developed SCAPE in the setting of underlying hypertension and multivalvular heart disease (severe MS, AS, and mild MR) with a significant hemodynamic alteration.

SCAPE can be diagnosed clinically. The patient usually presents with an acute onset of shortness of breath, which can advance into life‑threatening pulmonary edema usually within six hours in the absence of inadequate treatment [1,5]. In addition, systolic blood pressure is usually significantly elevated [5,6]. The findings of the chest X-ray could be bilateral infiltrations, Kerley B lines, and pleural effusion [2], which was seen in the chest X-ray of our patient at the presentation.

The presentation of SCAPE is a medical emergency that requires prompt diagnosis and adequate treatment [7]. In addition, immediate ED management of severe pulmonary edema decreases the rates of subsequent invasive mechanical ventilation and prolonged hospital stay [1].

The most critical intervention is BiPAP or continuous positive airway pressure (CPAP) [1]. Screening for the contraindication for the use of non-invasive ventilation (NIV) should be done rapidly. NIV can be provided using nasal or oronasal masks. Two common modes of NIV are CPAP and BiPAP.

The other main treatment goal is rapidly reducing blood pressure [2] and a common target for blood pressure is quickly reducing the systolic blood pressure to <140 mmHg [1,4,8]. The first line of treatment to control blood pressure is nitroglycerine infusion [9]. The nitroglycerine can be used sublingually until intravenous access is gained [1]. The American College of Emergency Physicians' clinical policy on AHFS recommends treating patients with AHFS and dyspnea with intravenous nitrates and CPAP [10]. Our patient well tolerated the therapy and improved with BiPAP and nitroglycerin.

## Conclusions

This case report has highlighted that SCAPE is an acute emergency requiring prompt diagnosis and management and the need to differentiate it from respiratory failure due to other causes. Urgent management with high-dose nitrates and NIV will prevent the need for endotracheal intubation and ICU admission requiring prolonged hospitalization. In addition, alteration in normal hemodynamics and the development of SCAPE could be because of chronic left ventricular failure due to valvular heart disease, as in our patient.
